# Prevalence and risk factors for carriage of antimicrobial-resistant *Escherichia coli* on household and small-scale chicken farms in the Mekong Delta of Vietnam

**DOI:** 10.1093/jac/dkv053

**Published:** 2015-03-08

**Authors:** Vinh Trung Nguyen, Juan J. Carrique-Mas, Thi Hoa Ngo, Huynh Mai Ho, Thanh Tuyen Ha, James I. Campbell, Thi Nhung Nguyen, Ngoc Nhung Hoang, Van Minh Pham, Jaap A. Wagenaar, Anita Hardon, Quoc Hieu Thai, Constance Schultsz

**Affiliations:** 1 Department of Medical Microbiology, Academic Medical Center, University of Amsterdam, Amsterdam, The Netherlands; 2 Department of Global Health-Amsterdam Institute for Global Health and Development, Academic Medical Center, University of Amsterdam, Amsterdam, The Netherlands; 3 Oxford University Clinical Research Unit, Centre for Tropical Medicine, Ho Chi Minh City, Vietnam; 4 Centre for Tropical Medicine, Nuffield Department of Medicine, University of Oxford, Oxford, UK; 5 Sub-Department of Animal Health, My Tho, Tien Giang, Vietnam; 6 Department of Infectious Diseases and Immunology, Faculty of Veterinary Medicine, Utrecht University, Utrecht, The Netherlands; 7 Central Veterinary Institute of Wageningen UR, Lelystad, The Netherlands; 8 Center for Social Science and Global Health, University of Amsterdam, Amsterdam, The Netherlands

**Keywords:** antimicrobial use, antimicrobial resistance, poultry, treatment incidence

## Abstract

**Objectives:**

To describe the prevalence of antimicrobial resistance among commensal *Escherichia coli* isolates on household and small-scale chicken farms, common in southern Vietnam, and to investigate the association of antimicrobial resistance with farming practices and antimicrobial usage.

**Methods:**

We collected data on farming and antimicrobial usage from 208 chicken farms. *E. coli* was isolated from boot swab samples using MacConkey agar (MA) and MA with ceftazidime, nalidixic acid or gentamicin. Isolates were tested for their susceptibility to 11 antimicrobials and for ESBL production. Risk factor analyses were carried out, using logistic regression, at both the bacterial population and farm levels.

**Results:**

*E. coli* resistant to gentamicin, ciprofloxacin and third-generation cephalosporins was detected on 201 (96.6%), 191 (91.8%) and 77 (37.0%) of the farms, respectively. Of the 895 *E. coli* isolates, resistance to gentamicin, ciprofloxacin and third-generation cephalosporins was detected in 178 (19.9%), 291 (32.5%) and 29 (3.2%) of the isolates, respectively. Ciprofloxacin resistance was significantly associated with quinolone usage (OR = 2.26) and tetracycline usage (OR = 1.70). ESBL-producing *E. coli* were associated with farms containing fish ponds (OR = 4.82).

**Conclusions:**

Household and small farms showed frequent antimicrobial usage associated with a high prevalence of resistance to the most commonly used antimicrobials. Given the weak biocontainment, the high prevalence of resistant *E. coli* could represent a risk to the environment and to humans.

## Abstract (precis)

*Escherichia coli* multidrug resistant to widely available antibacterials poses a threat to humans, their poultry and their environment when the prevalence is high, and containment is low.

## Introduction

Antimicrobials are extensively used in animal farming with the aim of treating and preventing animal diseases, as well as improving growth performance.^1^ The overuse of antimicrobials in food-animal farming is an important factor contributing to the emergence and dissemination of antimicrobial-resistant organisms in animal production systems, and contributes at an unknown level to the overall problem of antimicrobial resistance (AMR) in human medicine.^2^ The use of fluoroquinolones, aminoglycosides and third-generation cephalosporins in animal farming is of particular concern, since these are among the most important antimicrobials currently available to treat serious human infections.^3^

Commensal *Escherichia coli* organisms are commonly used to monitor the prevalence of AMR in livestock and poultry, since they reflect well the selective pressure on Gram-negative enteric bacteria.^4,5^ AMR determinants present in *E. coli* that are selected or amplified on farms may spread to humans through direct contact, by the consumption of meat or indirectly through environmental pathways.^6^ Furthermore, some animal-derived *E. coli* strains can also be pathogenic to humans or may act as a donor of AMR genes to other pathogenic Enterobacteriaceae.^7,8^

A number of studies have demonstrated an overall higher prevalence of AMR among chicken *E. coli* compared with human *E. coli* isolates^7,9^ and have incriminated chickens as a source of fluoroquinolone-resistant, extraintestinal pathogenic *E. coli* infections in humans.^7,10^ Because of this, the recently observed increase in plasmid-mediated resistance to fluoroquinolones among *E. coli* of chicken origin is of concern.^5,11^ Human infections caused by microorganisms that are resistant to third- and fourth-generation cephalosporins owing to the acquisition of ESBL genes have increased rapidly worldwide since they were first described in 1989. Recent reports have shown the presence of ESBL-producing *E. coli* in poultry^12–14^ and a great level of molecular similarity between ESBL-producing *E. coli* from chicken meat and humans, suggesting that chickens are a major source of this.^15–17^ A rise in aminoglycoside resistance in Gram-negative microorganisms has been described in European and Asian countries.^18^ In Vietnam, antimicrobials including fluoroquinolones and aminoglycosides are extensively used in large-scale pig and poultry farming,^19–21^ and a high prevalence of AMR to the two classes of antimicrobials has been observed in both commensal and zoonotic bacteria from farms and meat.^22,23^

Vietnam is an agricultural country with around 70% of the population living in rural areas. Around 40% of households engage in poultry-raising,^24^ and 94% of these 8 million households have a flock size of <50 chickens.^25^ Little is known about the prevalence of AMR in *E. coli* in such relatively small production systems or about its potential association with antimicrobial use and other farming practices. It is often assumed that, compared with larger farms, backyard farms use fewer antimicrobial drugs and more often feed their chickens with by-products instead of (often medicated) commercial feed. We therefore carried out a survey to investigate the prevalence of AMR in *E. coli* indicator bacteria in Vietnamese household and small chicken farms, with the aims of: (i) estimating the prevalence of *E. coli* resistant to key antimicrobials, with a focus on fluoroquinolones, aminoglycosides and third-generation cephalosporins; and (ii) identifying risk factors for the faecal carriage of antimicrobial-resistant *E. coli* in chickens, including demographics, management practices and antimicrobial usage.

## Methods

### Study population

With an area of 2481 km^2^, the province of Tien Giang (Vietnam) is home to ∼1.67 million people and ∼5.96 million chickens. For logistical reasons, the study was conducted in 3 districts (My Tho, Cho Gao and Chau Thanh) out of the 10 in the province as they contain 44.5% of the total chicken population of the province. The study population consisted of 208 chicken farms, equally divided into two strata according to the number of chickens per farm: ≥10–200 (‘household’ farms) and >200–2000 (‘small’ farms, in contrast to large scale farms with >2000 chickens). To avoid regional biases in the sampling, 34 farms from each of the four strata (district-farm size combinations) in Cho Gao and My Tho and 36 farms from each of the two strata in Chau Thanh were selected.

The number of farms to be sampled from each commune (the lower administrative unit within a district) was calculated with a probability that was directly proportional to the number of farms in that commune according to the Vietnamese rural, agricultural and fishery census in 2006.^26^ Farms were randomly sampled from each chosen commune. Farmers refusing to participate were replaced by the next eligible farm.

Written informed consent was obtained from all farmers prior to participation in the study. The study was approved by the Sub-Department of Animal Health (SDAH) and the Peoples' Committee of Tien Giang Province.

### Data collection

The farm visits were evenly distributed over the period March 2012 to April 2013 to avoid seasonal effects. Data on antimicrobial usage and farm management practices were collected using a structured questionnaire, which was conceived in a workshop including local facilitators and was tested in the field prior to sampling (available as Supplementary data at *JAC* Online). The questionnaire was aimed at the person with primary responsibility for chicken husbandry and contained both open and closed questions. This person was asked about details of the administration of any antibacterial formulation from restocking until the date of the visit for farms applying all-in-all-out (AIAO) systems, and for a fixed period of 90 days for the remaining farms not practising AIAO.

SDAH staff gathered data on each antibacterial formulation administered (excluding coccidiostats and antiparasitic and antifungal drugs), including the commercial name of the product, the presentation and the number of containers used. To facilitate the farmers' recall, open discussions were initiated after inspecting the medicine cabinet for all products present that contained antibacterial formulations. This approach is analogous to the medicine cabinet survey used in human medicine, which has been shown to be highly effective in obtaining information on the community usage of antimicrobial drugs.^27^

### Sample collection

From each flock, naturally pooled chicken faeces were collected from representative sections of the chicken pens/houses using two (household farms) or three (small farms) pairs of boot swabs attached to footwear. For unconfined flocks, boot swab samples were collected from the areas where the chickens roosted at night. Boot swabs were used to walk at least 30 steps on areas where fresh droppings were visible. For flocks housed on stilts or caged flocks where it was not possible to use boot swabs, visible faecal material was collected using two to three hand-held gauze swabs, which were similar in size to the boot swabs, each collecting material from at least 10 different locations.

The swab samples were immediately stored at 4°C, transferred to the laboratory in Ho Chi Minh City and cultured within 24 h after sample collection. Both the interviews and the faecal sample collection were conducted by trained veterinarians from Tien Giang SDAH.

### E. coli isolation

A fixed volume (225 mL) of buffered peptone water was added to each gauze or boot swab in a separate container and was then manually shaken. A volume of 1 mL from each container was pipetted and pooled into a sample. From this pooled sample, 1 mL was further diluted 1: 1000 in saline solution, and 50 μL of this suspension was plated onto MacConkey agar without supplement and MacConkey agar supplemented with ceftazidime (2 mg/L) to select for isolates with reduced susceptibility to third-generation cephalosporins, nalidixic acid (16 mg/L) to select for isolates with reduced susceptibility to quinolones, or gentamicin (8 mg/L) to select for isolates with reduced susceptibility to gentamicin. This was then incubated at 37°C overnight. The total number of suspect *E. coli* colonies was counted for each plate. A random selection of five (MacConkey agar not supplemented) and two (MacConkey agar supplemented with antimicrobial drugs) presumptive *E. coli* colonies of different morphologies were subcultured and were identified as *E. coli* using standard biochemical tests (hydrogen sulphide production, carbohydrate fermentation, urease test, nitrate reductase test, methyl red test, motility test and indole test) and/or API 20E (bioMérieux, France). Isolates confirmed as *E. coli* were tested for their antimicrobial susceptibility.

### Antimicrobial susceptibility testing

For the determination of antimicrobial susceptibility, the disc diffusion method was performed and interpreted according to breakpoints as defined by the CLSI.^28^ The following antimicrobials were tested at the given disc content: ampicillin (10 μg), ceftriaxone (30 μg), ceftazidime (30 μg), amoxicillin/clavulanic acid (30 μg), chloramphenicol (30 μg), ciprofloxacin (5 μg), trimethoprim/sulfamethoxazole (1.25/23.75 μg), gentamicin (10 μg), amikacin (30 μg), tetracycline (30 μg) and meropenem (10 μg). The potential production of ESBLs, as indicated by resistance to ceftriaxone and/or ceftazidime and by an inhibitory effect of clavulanic acid, was confirmed using a double disc diffusion test according to CLSI guidelines. Strains with an intermediate susceptible result were considered resistant. An MDR strain was defined as a strain resistant to at least three different classes of antimicrobials. A farm was defined as ‘positive’ for a resistant *E. coli* if at least one *E. coli* isolate resistant to the antimicrobial drug under study was cultured from MacConkey agar either with or without supplementation with antimicrobial drugs. Quality controls for identification and susceptibility testing were performed on a weekly basis according to CLSI guidelines.

Since all the MacConkey agar plates (i.e. with or without supplementation with antimicrobial drugs) were streaked using an identical inoculum, the counts of *E. coli*-like colonies on each plate were used to determine the proportion of colonies resistant to ceftazidime, gentamicin and nalidixic acid in relation to the total *E. coli* population for each farm.

### Data analyses

Since the study was designed as a stratified survey with a fixed number of farms in each stratum, not all the study units (farms) had the same probability of being selected. The prevalence of resistance to each antimicrobial of a randomly selected isolate cultured from non-selective plates, as well as the prevalence of resistance by farm, was adjusted for the stratified survey design by assigning a stratum-specific sampling weight (*W_i_*) to each observation unit (either isolate or farm) using the following equation: *W_i_* = *N_T_/N_i_,* where *N_T_* is the total number of farms in the three study districts (29 106) and *N_i_* is the number of farms in each stratum sampled (*i* = 1 … 6). Standard errors were corrected to take into account potential similarities of prevalence between the farms in each stratum.^29^

The frequency of antimicrobial treatment was quantified by calculating the treatment incidence (TI) as previously described.^30^ The TI is defined as the number of chickens per 1000 that are treated daily with one DDD of each antimicrobial administered on each farm using the following formula:

$$\begin{array}{l} \text{TI = }\frac{\text{ [total amount of antimicrobial administered (mg)]}}{\text{[DDDs (mg/kg)}\;\times \;\text{number of days at risk }}\\ \;\;\times \text{total weight of chickens on farm (kg)]} \end{array}$$

The total amount of an antimicrobial administered was calculated using: (i) the total consumption as reported by the farmer (i.e. the number of containers of antimicrobial-containing products used); (ii) the concentration of the product; and (iii) the reporting usage period.

The animal DDD was estimated based on the dosage mentioned in the drug's instruction leaflet. In case the medication was dissolved in drinking water or feed, the dosage as indicated by the manufacturer was standardized to mg/kg chicken body weight, given that an average chicken consumes 190 mL of water and 80 g of feed per day. The average weight of one chicken was considered to be 1 kg.^31^ The Anatomical Therapeutic Chemical classification system for veterinary medicinal products (ATCvet)^32^ was used for antimicrobial drug identification.

To determine the risk factors associated with resistance that are considered of clinical importance for human medicine, we modelled the probability of a randomly selected *E. coli* isolate from any given farm for the following three outcomes: (i) resistance to ciprofloxacin; (ii) resistance to gentamicin; and (iii) multidrug resistance. This was carried out by building hierarchical generalized linear mixed regression models with the term ‘farm’ modelled as a random effect.

For the outcome ‘resistance to third-generation cephalosporins’, where we observed a very low probability of resistance among individual randomly selected *E. coli* isolates (3.2%), culture results from supplemented and unsupplemented plates were combined and standard logistic regression models were built to model the probability of the presence of resistant strains on the farm.

To build each model, a total of 42 variables were first tested in univariable analyses, including factors describing the farms (production type, size and presence of other animals), farmers' demographic factors, husbandry factors and antimicrobial usage (see the Supplementary data for all the variables that were included). Variables were considered as a candidate for multivariable analysis based on their biological plausibility and a *P* value <0.15 in the univariable analyses. Candidate variables were ranked by their degree of significance and were included in the models starting with the most significant and using a stepwise forward approach.^33^ In the final multivariable models, variables were retained if their *P* value was <0.05. All interactions between all significant variables in the model were assessed.

All statistical analyses were performed using the packages *epicalc* and *survey* with R statistical software (http://www.r-project.org).

## Results

### Description of farm demographic and management factors

Of the 104 household farms, 76.0% raised chickens for meat, whereas 23.1% raised chickens with a mixed purpose (meat and eggs). In contrast, 60.6% of the 104 small farms raised egg-laying flocks and 38.5% raised meat chickens (Table 1). The confinement of chickens in pens or houses for 24 h per day was more common in small farms compared with household farms (89.4% versus 1.9%, respectively) (*P *< 0.001). The percentage of small farms that used commercial feed (99.0%) was greater than the percentage of household farms that followed this practice (70.2%) (*P *< 0.001).

**Table 1.** Characteristics of 208 chicken farms in Tien Giang province, Vietnam, studied between March 2012 and April 2013

Variable	Household farms (*n* = 104)	Small farms (*n* = 104)
Age of farm manager (years), median (IQR)	46 (40–55)	43 (37–52)
Male farm manager, no. of farms (%)	59 (56.7)	77 (74.0)
Level of education attained, no. of farms (%)		
up to primary school	38 (36.5)	18 (17.3)
secondary school	40 (38.5)	54 (51.9)
higher	26 (25.0)	32 (30.8)
No. of chickens, median (IQR)	75 (63–120)	1500 (1000–1900)
Production type, no. of farms (%)		
meat	79 (76.0)	40 (38.5)
eggs	1 (1.0)	63 (60.6)
mixed purpose	24 (23.1)	1 (1.0)
Age of chickens (weeks), median (IQR)	15 (8–20)	20 (8–32)
AIAO system, no. of farms (%)	32 (30.8)	68 (65.4)
Chickens confined in pen/house 24 h per day, no. of farms (%)	2 (1.9)	93 (89.4)
Source of day-old chickens, no. of farms (%)		
hatched on farm	59 (58.4)	10 (11.2)
local hatchery	23 (22.8)	19 (21.3)
company hatchery	8 (7.9)	59 (66.3)
other	11 (10.9)	1 (1.1)
Presence of animals other than chickens, no. of farms (%)	103 (99.0)	97 (93.3)
duck(s)	47 (45.2)	27 (26.0)
pig(s)	54 (51.9)	42 (40.4)
cattle/buffalo(s)	22 (21.2)	15 (14.4)
dog(s)	97 (93.3)	83 (79.8)
cat(s)	58 (55.8)	54 (51.9)
fish/fish pond(s)	65 (62.5)	54 (51.9)
Change shoes/boots before entering pen/house, no. of farms (%)	53 (51.0)	90 (86.5)
Foot bath/foot dip at entrance, no. of farms (%)	43 (41.3)	82 (78.8)
Use of commercial feed, no. of farms (%)	73 (70.2)	103 (99.0)
Use of antimicrobials, no. of farms (%)	49 (47.1)	72 (69.2)

### Prevalence of AMR in E. coli isolates

A total of 895 *E. coli* isolates were recovered from unsupplemented MacConkey agar. The crude (unadjusted) and adjusted prevalence of resistance in the *E. coli* isolates are presented in Table 2. Among these randomly selected *E. coli* isolates, the adjusted prevalence of resistance to ciprofloxacin was 24.2% (Table 2). The adjusted prevalence of resistance to gentamicin was 15.0% and to ‘any third-generation cephalosporin’ (ceftazidime and/or ceftriaxone) was 3.1% (Table 2). A total of 81.3% of isolates were MDR (Table 2).

**Table 2.** Prevalence of AMR in *E. coli* isolates and on chicken farms without and with sampling adjustment in Tien Giang province, Vietnam

Antimicrobial	*E. coli* isolates^a^ (*n* = 895)		Farms^b^ (*n* = 208)	
	prevalence of resistance (%)	adjusted prevalence (%) (95% CI)	prevalence of resistance (%)	adjusted prevalence (%) (95% CI)
Tetracycline	93.4	91.1 (88.4–93.7)	100	100 (100–100)
Trimethoprim/sulfamethoxazole	69.7	67.0 (62.7–71.3)	100	100 (100–100)
Chloramphenicol	68.1	61.2 (57.1–65.4)	99.0	100 (99.9–100)
Gentamicin	19.9	15.0 (11.8–18.1)	96.6	98.2 (95.0–100)
Amikacin	5.4	5.4 (3.5–7.4)	22.1	22.3 (13.1–31.5)
Ciprofloxacin	32.5	24.2 (20.3–28.1)	91.8	92.8 (87.2–98.4)
Ampicillin	86.0	83.2 (79.5–87.0)	100	100 (100–100)
Amoxicillin/clavulanic acid	47.9	44.2 (39.6–48.9)	95.7	95.0 (89.7–100)
Ceftazidime	2.0	1.9 (0.4–3.5)	31.2	44.2 (33.1–55.3)
Ceftriaxone	2.5	2.2 (0.7–3.7)	35.1	44.6 (33.5–55.7)
Third-generation cephalosporins^c^	3.2	3.1 (1.3–4.9)	37.0	45.9 (34.8–57.0)
ESBL-confirmed	0.2	0.4 (0–1.1)	14.9	20.6 (11.5–29.7)
Meropenem	0	0	0	0
MDR^d^	85.3	81.3 (77.8–84.8)	100	100 (100–100)

^a^Prevalence of resistance among *E. coli* isolates randomly picked from unsupplemented MacConkey agar plates representing an unbiased snapshot of the *E. coli* population.

^b^Prevalence of resistance among chicken farms based on the isolation of resistant *E. coli* using selective MacConkey agar containing ceftazidime, gentamicin or nalidixic acid.

^c^Ceftazidime and/or ceftriaxone.

^d^Resistant to at least three different classes of antimicrobial drugs.

### Prevalence of antimicrobial-resistant E. coli on chicken farms

*E. coli* isolates resistant to tetracycline, trimethoprim/sulfamethoxazole, chloramphenicol and ampicillin were detected on 100% of farms. Isolates resistant to gentamicin (98.2%), amoxicillin/clavulanic acid (95.0%) and ciprofloxacin (92.8%) were also prevalent at most farms, whereas isolates resistant to ceftriaxone (44.6%), ceftazidime (44.2%) and amikacin (22.3%) were less common. At least one ESBL-producing *E. coli* isolate was recovered from 20.6% of farms. MDR *E. coli* isolates were identified at all farms (Table 2).

### Proportion of E. coli isolates showing resistance by farms

**Figure 1.**
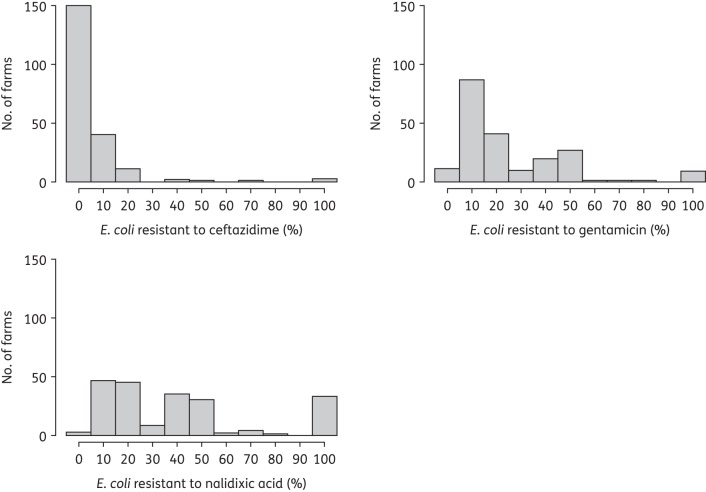
Distribution of the percentage of *E. coli* isolates resistant to ceftazidime, gentamicin and nalidixic acid across all farms (*n* = 208).

The proportion of *E. coli* isolates that were resistant to ceftazidime, gentamicin and nalidixic acid in relation to the total *E. coli* population in each farm was estimated and is depicted in Figure 1*.* Colonies resistant to gentamicin and nalidixic acid accounted for 100% of *E. coli*-like colonies for 9 (4.3%) and 32 (15.4%) farms, respectively.

### Antimicrobial usage

The TIs of different classes of antimicrobial drugs are shown in Table 3. The mean TI was highest for tetracyclines (90.8) followed by macrolides (73.3), penicillins (52.1) and polymyxins (51.3) (Table 3). The TI for overall antimicrobial drug consumption was 370.6, meaning that, on average per day, 371 chickens out of 1000 were treated with one DDD of an antimicrobial drug.

**Table 3.** TI of different classes of antimicrobial drugs used on household and small-scale chicken farms in Tien Giang province, Vietnam (*n* = 208)

Class of antimicrobial drug^a^	Name of antimicrobial drug	No. of farms using antimicrobial	Mean TI	Standard deviation
Tetracyclines	doxycycline, oxytetracycline, tetracycline	52	90.8	608.9
Macrolides	tylosin, tilmicosin, erythromycin, spiramycin	40	73.3	582.0
Polymyxins	colistin	39	51.3	234.2
Penicillins	ampicillin, amoxicillin	33	52.1	383.1
Quinolones	flumequine, oxolinic acid, norfloxacin, enrofloxacin	19	44.3	304.9
Aminoglycosides	neomycin, gentamicin, apramycin, streptomycin	15	8.0	40.7
Amphenicols	florfenicol, thiamphenicol	13	6.4	54.2
Sulphonamides	sulfamethoxazole, sulfadimidine, sulfadimethoxine, sulfamerazine	10	15.5	140.6
Lincosamides	lincomycin	4	8.5	81.9
Spectinomycin	spectinomycin	4	10.0	85.0
Trimethoprim	trimethoprim	2	0.3	2.9
Pleuromutilins	tiamulin	1	0.1	1.0
All classes	all antimicrobials	121	370.6	1447.4

^a^Classes were based on ATCvet classification.

### Risk factor analyses

The use of quinolones (OR = 2.26) and tetracyclines (OR = 1.70) was significantly associated with ciprofloxacin resistance in *E. coli* isolates (Table 4). A small farm size and farming strategies including the use of commercial feed, the non-practising of an AIAO system and a change shoes/boots practice were all associated with ciprofloxacin resistance, but these associations were not independent (Table 4). We observed significant interactions between small farm size and the practice of changing shoes/boots (OR = 0.22) as well as between the usage of commercial feed and use of the AIAO method (OR = 10.99).

**Table 4.** Risk factors for resistance to ciprofloxacin, resistance to gentamicin and multidrug resistance in 895 randomly selected *E. coli* isolates recovered from 208 chicken farms (Tien Giang province, Vietnam)

Outcome	Variable	OR	95% CI	*P*
Ciprofloxacin resistance^a^	small farm (baseline = household farm)	6.42	2.74–15.03	<0.001
	use of commercial feed	1.87	1.06–3.30	0.032
	change shoes/boots practice	2.43	1.44–4.09	<0.001
	AIAO system	0.17	0.02–1.28	0.086
	use of quinolones	2.26	1.20–4.25	0.011
	use of tetracyclines	1.70	1.05–2.76	0.031
	interaction ‘small farm’ and ‘change shoes/boots’	0.22	0.09–0.55	0.001
	interaction ‘use of commercial feed’ and ‘AIAO’	10.99	1.38–87.7	0.024
Gentamicin resistance^b^	use of tetracyclines	1.99	1.17–3.36	0.011
	presence of cat(s)	0.44	0.24–0.82	0.010
	change shoes/boots practice	2.41	1.27–4.59	0.007
	day-old chickens from other sources^c^	4.93	1.22–19.97	0.026
	use of lincosamides	4.74	1.18–18.97	0.028
	log(density)^d^	1.32	1.02–1.69	0.034
	chicken purpose (baseline = egg-laying chicken)			
	meat chicken	9.88	5.32–18.33	<0.001
	mixed chicken	5.03	1.81–14.01	0.002
Multidrug resistance^e,f^	use of commercial feed	2.49	1.14–4.14	0.001
	log(density)	1.28	1.06–1.54	0.008
	years of experience in chicken farming	0.96	0.93–0.99	0.004

^a^Intercept: −2.60 (SEM ± 0.28).

^b^Intercept: −5.79 (SEM ± 0.74).

^c^Baseline = day-old chickens from industrial hatchery companies. Other sources include local hatcheries, markets and neighbours.

^d^Number of chickens per m^2^.

^e^Intercept: 1.41 (SEM ± 0.28).

^f^Resistant to at least three different classes of antimicrobial drugs.

Lincosamide (OR = 4.74) and tetracycline (OR = 1.99) usage was associated with resistance to gentamicin in *E. coli* isolates. In addition, farming strategies, including a change shoes/boots practice (OR = 2.41), the purchase of day-old chickens from sources other than industrial hatchery companies (local hatcheries, markets, neighbours etc.) (OR = 4.93) and raising chickens for meat or mixed (meat and egg) purposes, but not solely for egg-laying purposes, (OR = 9.88 and OR = 5.03, respectively) were associated with the isolation of gentamicin-resistant *E. coli*. A high density of chickens (number of chickens per m^2^) was associated with both gentamicin resistance and multidrug resistance. We observed a 32% and 28% increase in the odds of isolating gentamicin-resistant or MDR *E. coli*, respectively, for a one unit increase in chicken density (chickens per m^2^). The use of commercial feed was also associated with the isolation of MDR *E. coli* (OR = 2.49). The risk of carriage of MDR *E. coli* was decreased by 4.0% for a one unit increase in the farmer's number of years of experience of chicken-farming.

The presence of fish pond(s) (OR = 2.93, 95% CI = 1.11–7.76) and the usage of any antimicrobial drug (OR = 2.80, 95% CI = 1.08–7.28) were associated with resistance to third-generation cephalosporins in *E. coli*. The presence of fish pond(s) (OR = 4.82, 95% CI = 1.27–18.27), the purchase of day-old chickens from other sources (local hatcheries, markets, neighbours etc.) compared with day-old chickens from industrial hatchery companies (OR = 13.02, 95% CI = 1.89–89.61) and having a change shoes/boots practice on the farm (OR = 3.4, 95% CI = 0.98–11.81) were associated with the presence of ESBL-producing *E. coli* on the farm.

## Discussion

This study demonstrated a very high (81.3%) prevalence of MDR *E. coli* isolated from household and small-scale chicken farms in an unbiased study population in the Mekong Delta of Vietnam. The prevalence of resistance to both ciprofloxacin (24.2%) and gentamicin (15.0%) was substantial, while resistance to third-generation cephalosporins (3.1%) was of a much lower level. The prevalence of resistance among chicken farms based on the isolation of resistant *E. coli* using selective culture media was very high (Table 2). Our results indicate a generally higher or similar prevalence of AMR among chicken *E. coli* isolates from Vietnam to commonly used antimicrobials (tetracycline, chloramphenicol, ampicillin and gentamicin) compared with results from industrialized countries.^34–36^ Data from seven European countries suggest a higher prevalence of ciprofloxacin resistance (57.6%), while data from five European countries indicate a higher prevalence of ceftazidime resistance (11.1%) in chickens in these countries.^37^ Although such comparisons should be interpreted with caution because of differences in sampling methods as well as differences in the breakpoints used for interpreting susceptibility test results between studies from different regions, the high prevalence of AMR observed in these backyard farms in Vietnam is striking and unexpected.

The observed high prevalence of AMR reflects the common use of antimicrobial products for therapeutic and prophylactic purposes, as found in our survey on antimicrobial drug usage. Even though there was a large variation in TI between farms and between antimicrobial drugs, the TI of any antimicrobial drug usage calculated in our study (370.6) was much higher than the TI calculated for countries with industrial broiler production such as Belgium (131.8), the Netherlands (82.2) and Denmark (8.2).^30,38^ However, such comparisons should be interpreted with caution given the differences in study design. In addition, most of these products were available without prescription in a pilot survey across 20 veterinary drug stores in the area (data not shown).

We found statistical associations between the usage of quinolones and tetracyclines and ciprofloxacin resistance, as well as between the usage of tetracyclines and lincosamides and resistance to gentamicin. Other field studies have also demonstrated that the use of quinolones selects for the carriage of quinolone-resistant *E. coli* in poultry.^4,39^ The association between tetracycline use and quinolone resistance may be explained by an effect of tetracycline-induced mutations in the *mar* operon resulting in an overexpression of MarA, which increases resistance to multiple drugs including quinolones.^40^ Finally, the co-selection of resistance determinants, encoded by genes located on mobile elements such as integrons, could explain the observed association between the usage of tetracyclines and lincomycin, which is often formulated in combination with spectinomycin, and resistance to gentamicin.^41^ We acknowledge the limitations in obtaining accurate usage data derived from a cross-sectional study design. Recall biases with regard to data on usage may have introduced error with an unknown impact on the observed associations. In addition, we tried to use the TIs of different antimicrobials as continuous variables in the risk factor analyses. However, we did not succeed in achieving a stable model with these continuous variables and as a result we had to consider them as binary variables for the analyses. Despite these limitations, our study provides a unique view on antimicrobial drug usage and associated AMR in backyard chicken farms in Vietnam.

The use of commercial feed was associated with an increased risk of fluoroquinolone resistance and multidrug resistance, in agreement with a study on turkey farms in Europe,^39^ and reflects the fact that in Vietnam commercial poultry feed is commonly medicated with antimicrobials.^42^ In this study, we randomly collected 25 feed samples from 25 different chicken farms and tested these for the presence of antimicrobial agents (Premi-Test, R-Biopharm AG). Antimicrobial compound(s) were detected in all the feed samples (data not shown). The test does not, however, allow a further identification of the antimicrobial compounds that were present or their concentrations in the feed.

Independent of antimicrobial drug or medicated feed usage, there was mixed evidence of an association between the intensification of chicken production and AMR. For example, *E. coli* isolates from household farms had clearly lower levels of ciprofloxacin resistance than isolates from small farms, and an increase in density of the chickens was associated with gentamicin resistance and multidrug resistance. In contrast, AIAO systems, which were more commonly observed at the larger farms, decreased the risk of ciprofloxacin resistance, while the purchase of day-old chickens from company hatcheries and the production of layer flocks were associated with lower levels of gentamicin resistance, in line with studies in Europe that have reported a much lower level of gentamicin resistance in layer chickens compared with broiler chickens.^37^

We did not find evidence of any usage of third-generation cephalosporins on any chicken farm surveyed. However, in Vietnam, cephalosporins are among the antimicrobial classes most commonly used in human medicine.^43,44^ It is therefore possible that there may have been a transmission of resistance determinants from humans or other species (e.g. pigs) to chickens, which would explain the observed, albeit low-prevalence, resistance to ceftazidime and ceftriaxone. We found that the presence of an integrated fish pond at a farm was associated with the isolation of third-generation cephalosporin-resistant and ESBL-producing *E. coli*. We speculate that this association was related to the contact of the chicken with fish pond water, which would underscore the relevance of human activities for AMR in poultry, since a relatively high proportion of households in the rural areas of the Mekong Delta do not have latrines that meet established hygiene standards in terms of their construction, operation and maintenance.^45^ A recent study in China suggested that the presence of ESBL-positive Enterobacteriaceae in fish farms was likely to have originated from contamination with human sewage.^46^ Further comparisons of isolates from humans, chickens and fish ponds should help to elucidate this relationship.

We have identified several potential risk factors for AMR in household and small-scale farms in southern Vietnam, which include antimicrobial usage, farm management practices and environmental risks. Given the existing low levels of ‘biocontainment’ on these farms and the rare use of personal protective equipment for farming personnel when dealing with the animals, as well as the fact that there is a great degree of overlap between the farming and the household environments, the risks of transmission of antimicrobial-resistant *E. coli* posed to both farmers and the communities living in the proximity of chicken farms are likely to be high. These need to be properly assessed in order to formulate effective strategies to limit the further development of resistance to safeguard human health.

## Funding

This work was funded by ZonMW/WOTRO (number 205100012), The Netherlands and the Wellcome Trust, UK (089276/Z/09/Z).

## Transparency declarations

None to declare.

## Supplementary data

Supplementary data are available at *JAC* Online (http://jac.oxfordjournals.org/).

Supplementary Data
