# Supplementary material for: Prevalence and risk factors for carriage of antimicrobial-resistant Escherichia coli on household and small-scale chicken farms in the Mekong Delta of Vietnam
Source: J Antimicrob Chemother. 2015 Mar 8;70(7):2144–52. doi: 10.1093/jac/dkv053 (PMC4472326; doi:10.1093/jac/dkv053)
Supplement: Supplementary Data [file supp_70_7_2144__index.html]

Prevalence and risk factors for carriage of antimicrobial-resistant Escherichia coli on household and small-scale chicken farms in the Mekong Delta of Vietnam — Supplementary Data 

# Prevalence and risk factors for carriage of antimicrobial-resistant *Escherichia coli* on household and small-scale chicken farms in the Mekong Delta of Vietnam

## Supplementary Data

Supplementary Data

**Files in this Data Supplement:**

- Supplementary Data - Supplementary Data
- Supplementary Data - Supplementary Data
