# Supplementary material for: Prevalence and risk factors for carriage of antimicrobial-resistant Escherichia coli on household and small-scale chicken farms in the Mekong Delta of Vietnam
Source: J Antimicrob Chemother. 2015 Mar 8;70(7):2144–52. doi: 10.1093/jac/dkv053 (PMC4472326; doi:10.1093/jac/dkv053)
Supplement: Supplementary Data [file supp_dkv053_dkv053supp1.doc]

Name of interviewer: [_________________________________]

Interview date (dd/mm/yy) : [__|__]/[__|__]/[__|__]

We are conducting a study to investigate medicines used by Tien Giang farmers for their chickens. You have received information about our study and you have agreed to participate. We would like to ask you some questions about your farm and your experience in the use of medicines for your chicken. For example, which medicines do you use and when and why. Do you agree to do the interview now?

| 1. **GENERAL INFORMATION** |
| --- |
| 1. Age of farm owner/manager (years): [__|__] years |
| 1. Gender: ⭘ Male ⭘ Female |
| 1. Highest educational attainment: ⭘ No schooling ⭘ Primary school ⭘ Secondary school ⭘ High school ⭘ Post-high school degree |
| 1. Years of experience in poultry farming: [__|__] years |
| 1. **PROFILE OF CHICKEN FLOCK(S):** |
| 1. Please provide details on chicken flock(s) present on your farm now   Use a different flock number for each poultry keeping area |

| **Flock number** | **Chicken purpose** | **Total number** | **Age** | **Are your birds confined 24 hours/day in a house/pen?** | **If confined, are they kept inside 24h/day** | **If unconfined or partly confined, do they have access to outside the farm?** | **All-in/all-out** | **No of crop/s per year** | **Expected age of depopulation or sale. If chicken are sold at different ages, indicate range** | **Chicken procured as** | | | |
| --- | --- | --- | --- | --- | --- | --- | --- | --- | --- | --- | --- | --- | --- |
|  |  |  |  |  |  |  |  |  |  | **Day-olds** | | **At age other than day-olds, specify age at purchase** | |
|  |  |  |  |  |  |  |  |  |  | 1 - Yes  2 - No | 1-Hatched in farm  2-Purchased from local hatchery  3-From company hatchery  4-Purchased from Market/ Dealer/ neighbor  9-Unknown | 1 - Yes  2 - No | (in weeks)  (99 if unknown) |
|  | 1-Meat chicken  2-Layer chicken  3-Mixed purpose chickens |  | (in week/s) | 0 - Unconfined  1 - Pen  2 - House | 1 - Yes  2 - No | 1 - Yes  2 - No | 1 - Yes  2 - No |  | (in weeks) |  |  |  |  |
| [__|__] | [__] | [__|__|__|__] | [__|__] | [__] | [__] | [__] | [__] | [__] | [__|__] | [__] | [__] | [__] | [__|__] |
| [__|__] | [__] | [__|__|__|__] | [__|__] | [__] | [__] | [__] | [__] | [__] | [__|__] | [__] | [__] | [__] | [__|__] |
| [__|__] | [__] | [__|__|__|__] | [__|__] | [__] | [__] | [__] | [__] | [__] | [__|__] | [__] | [__] | [__] | [__|__] |
| [__|__] | [__] | [__|__|__|__] | [__|__] | [__] | [__] | [__] | [__] | [__] | [__|__] | [__] | [__] | [__] | [__|__] |
| [__|__] | [__] | [__|__|__|__] | [__|__] | [__] | [__] | [__] | [__] | [__] | [__|__] | [__] | [__] | [__] | [__|__] |
| [__|__] | [__] | [__|__|__|__] | [__|__] | [__] | [__] | [__] | [__] | [__] | [__|__] | [__] | [__] | [__] | [__|__] |
| [__|__] | [__] | [__|__|__|__] | [__|__] | [__] | [__] | [__] | [__] | [__] | [__|__] | [__] | [__] | [__] | [__|__] |

| 1. Have any of the chickens currently present in your farm been vaccinated? 2. ⭘ Yes ⭘ No   If Yes, tick all that apply  🞏 HPAI 🞏 Newcastle 🞏 Gumboro 🞏 Infectious bronchitis  🞏 Infectious encephalomyelitis 🞏 Fowl 🞏 Cholera 🞏 Marek  🞏 Other | | | | |
| --- | --- | --- | --- | --- |
| 1. Please provide details on previous chicken crop(s) – the one(s) occupying chicken pens/houses immediately before the current one(s) | | | | |
| **Pen/House**  **Number (use same number as in q. 5)** | **No of chickens** | **Age of chickens at depopulation or**  **sale (in weeks)** | **How many were lost due to diseases?** | **How many were lost due to other causes?** |
|
| [__|__] | [__|__|__|__] | [__|__] | [__|__|__] | [__|__|__] |
| [__|__] | [__|__|__|__] | [__|__] | [__|__|__] | [__|__|__] |
| [__|__] | [__|__|__|__] | [__|__] | [__|__|__] | [__|__|__] |
| [__|__] | [__|__|__|__] | [__|__] | [__|__|__] | [__|__|__] |
| [__|__] | [__|__|__|__] | [__|__] | [__|__|__] | [__|__|__] |
| 1. Any there other animal species on the farm now? ⭘ Yes ⭘ No   If Yes, tick all that apply  🞏 Fighting cock 🞏 Duck 🞏 Muscovy duck 🞏 Other poultry species  🞏 Pigs 🞏 Cattle/buffalo 🞏 Dog 🞏 Cat 🞏 Fish | | | | |
| 1. **ANTIBIOTIC USE** | | | | |
| 1. What do you do to keep your chickens healthy? Probe, anything else?   [____________________________________________________________________]  [____________________________________________________________________] | | | | |
| 1. What products have you used to keep your chickens healthy? Do you keep them in the farm? Can we see them?   Which have you used in the current flock(s)? For continuously occupied houses or farms, describe use over the last 3 months.  If no antibiotic is used, tick this box 🞏 | | | | |

| **Flock number** | |  |  |  |  |  |
| --- | --- | --- | --- | --- | --- | --- |
| **Commercial Name** | |  |  |  |  |  |
| **Manufacturer** | |  |  |  |  |  |
| **Contents** | |  |  |  |  |  |
| **Supplier (1)** | |  |  |  |  |  |
| **Complete only for the current (crop)** | **Presentation (2)** |  |  |  |  |  |
|  | **Content per unit (eg. Grams of active compound)** |  |  |  |  |  |
|  | **Administration (3)** |  |  |  |  |  |
|  | **Total number of units used on the flock**  **(probe, if don’t remember write dk)** |  |  |  |  |  |
|  | **How long ago was the last administration? (days) (probe, if don’t remember write dk)** |  |  |  |  |  |
|  | **Purpose of use (4)** |  |  |  |  |  |
|  | **Diseases/Problems (5)** |  |  |  |  |  |
|  | **Advice from (6)** |  |  |  |  |  |
|  | **Timing of application (7)** |  |  |  |  |  |

Coding for products: see product list and pictures

| ^(1)^ Supplier | 1- Drug/feed shop; 2- Drug company/salesman; 3-Friend/neighbor; 9-Other, if applicable, specify |
| --- | --- |
| ^(2)^ Formulation | 1- Powder; 2- Liquid |
| ^(3)^ Administration | 1-Dissolve in drinking water; 2-Mix with feed; 3- Both dissolve in drinking water and mix with feed; 4- Injection; 5-Nose drops |
| ^(4)^ Purpose of use | 1-Prevention; 2- Treatment; 3- Both prevention and treatment; 9- Other, specify |
| ^(5)^ Symptom | 1-Respiratory problems; 2-Digestive problems; 3-Poor performance/Malaise; 4-High mortality; 9-Other, if applicable, write down symptoms:…………………….. |
| ^(6)^ Advice from | 1-Drug seller; 2-District veterinarian; 3-Chief of animal health worker; 4-Salesperson; 6-Friend/neighbor; 9-Other, if applicable, write down advisor ……………… |
| ^(7)^ Timing of application | 1-On arrival; 2-Before/after vaccination; 3-Changing of feed; 4-Changing of season; 5-Before selling; 6-Other, if applicable, write down timing…………………… |

| 1. Do you read the administration guidelines of the antibiotics before use?   ⭘ Always ⭘ Sometimes ⭘ Never |
| --- |
| 1. **BIO-SECURITY AND CLEANING & DISINFECTION (C&D) OF CHICKEN HOUSES:** |
| 1. Ask only for enclosed chicken house/pen. Tick those that apply to your chicken flock(s):   If no chicken house/pen tick box 🞏 |

| **Flock number** | **Ante-room** | **Change of boot/shoes** | **Foot bath/boot dip** | **Are outsiders allowed?** |
| --- | --- | --- | --- | --- |
| [__|__] | ⭘ Yes ⭘ No | ⭘ Yes ⭘ No | ⭘ Yes ⭘ No | ⭘ Yes ⭘ No |
| [__|__] | ⭘ Yes ⭘ No | ⭘ Yes ⭘ No | ⭘ Yes ⭘ No | ⭘ Yes ⭘ No |
| [__|__] | ⭘ Yes ⭘ No | ⭘ Yes ⭘ No | ⭘ Yes ⭘ No | ⭘ Yes ⭘ No |
| [__|__] | ⭘ Yes ⭘ No | ⭘ Yes ⭘ No | ⭘ Yes ⭘ No | ⭘ Yes ⭘ No |
| [__|__] | ⭘ Yes ⭘ No | ⭘ Yes ⭘ No | ⭘ Yes ⭘ No | ⭘ Yes ⭘ No |

1. Ask only for enclosed chicken house/pen. Please describe the procedure of cleaning and disinfection in the chicken house/pen(s). Tick those that apply to your chicken flock(s):

If no chicken house/pen tick box 

| **Flock number** | **Type of C&D** | **Mucking out** | **Washing** | **Disinfec-tion** | **What do you do with the used muck/litter/bedding?** | **If Dispose** |
| --- | --- | --- | --- | --- | --- | --- |
|  | 1 - During production  2 – Terminal  3- Both 1 and 2  4-None | 1- Yes  2-No | 1- Yes  2-No | 1- Yes  2-No | 1-Fertilize your field  2-Dispose  3-Sell it | 1-Water way  2-Burn  3-Others |
|  |  |  |  |  |  |  |
| [__|__] | **[__]** | **[__]** | **[__]** | **[__]** | **[__]** | **[__]** |
| [__|__] | **[__]** | **[__]** | **[__]** | **[__]** | **[__]** | **[__]** |
| [__|__] | **[__]** | **[__]** | **[__]** | **[__]** | **[__]** | **[__]** |
| [__|__] | **[__]** | **[__]** | **[__]** | **[__]** | **[__]** | **[__]** |
| [__|__] | **[__]** | **[__]** | **[__]** | **[__]** | **[__]** | **[__]** |
| [__|__] | **[__]** | **[__]** | **[__]** | **[__]** | **[__]** | **[__]** |

1. Ask only for enclosed chicken house/pen. What disinfectants do you use for cleaning and disinfection of your chicken house?

If no chicken house/pen tick box 🞏 If no disinfectant is used, tick this box🞏.

| **Flock number** | **Commercial name of disinfectants** | **Dilution rate** | **Method /Application**  1-Pressure washer  2- Sprayer  4-Backpack  5-Hose  6-Others |
| --- | --- | --- | --- |
| [__|__] |  |  | [__] |
| [__|__] |  |  | [__] |
| [__|__] |  |  | [__] |
| [__|__] |  |  | [__] |
| [__|__] |  |  | [__] |

1. Wild birds seen in the farm

⭘ Never ⭘ Sometimes (1-4 times per month) ⭘ Often (>4 times per month)

1. Rodents seen in the farm

⭘ Never ⭘ Sometimes (1-4 times per month) ⭘ Often (>4 times per month)

1. Do you use any other product for disinfection? ⭘ Yes ⭘ No

a. If yes, specify: [________________________________________________________]

1. **CHICKEN FEED**
2. Please describe the types of feed you give to your chickens. Include feed you have given in your farm to previous flock(s) or the current chicken flock(s) only. Do not include any feed you intend to give in the future.

| **Flock number** | **Household left-overs** | **Uncooked rice/rice by products** | **Locally mixed chicken feed** | **Commercial feed** | **Others, specify** |
| --- | --- | --- | --- | --- | --- |
| [__|__] | 🞏 | 🞏 | 🞏 | 🞏 |  |
| [__|__] | 🞏 | 🞏 | 🞏 | 🞏 |  |
| [__|__] | 🞏 | 🞏 | 🞏 | 🞏 |  |
| [__|__] | 🞏 | 🞏 | 🞏 | 🞏 |  |
| [__|__] | 🞏 | 🞏 | 🞏 | 🞏 |  |

1. Please provide details on commercial feed given to the current crop(s) until present.

If no commercial feed is given, tick this box .

| **Flock number** | **Commercial name** | **Manufacturer** | **Presentation**  1-Crumbs  2-Pellet  3-Mash | **Quantity**  **(in kg)** | **Given to previous crop(s)**  1-Yes  2-No |
| --- | --- | --- | --- | --- | --- |
| [__|__] |  |  | [__] |  | [__] |
| [__|__] |  |  | [__] |  | [__] |
| [__|__] |  |  | [__] |  | [__] |
| [__|__] |  |  | [__] |  | [__] |
| [__|__] |  |  | [__] |  | [__] |

1. Do you use other products for your chicken, for example additives to feed or drinking water?

⭘ Yes ⭘ No

If yes, list: [_________________________________________________________]

1. Source of water for chickens (tick all that apply)

🞏 Municipal supply 🞏 Borehole/well 🞏 Rain water

🞏 Pond 🞏 River/stream/canal 🞏 other, specify________________

1. Distance from the farm to the closest running water sources (in meter) [__|__|__|__|__]

| 1. **QUESTION** |
| --- |
| 1. Do you have any questions or comments? ⭘ Yes ⭘ No   [_____________________________________________________________________]  [_____________________________________________________________________] |

Thank you, this is the end of the interview.
